# Enhancement of Ovarian Reserve and Oocyte Quality After Platelet-Rich Plasma Instillation in a Woman With Diminished Anti-Müllerian Hormone

**DOI:** 10.7759/cureus.53474

**Published:** 2024-02-02

**Authors:** Jarul Shrivastava, Akash More, Virul Shrivastava, Namrata Choudhary, Deepti Shrivastava

**Affiliations:** 1 Clinical Embryology, Datta Meghe Institute of Higher Education & Research, Wardha, IND; 2 Obstetrics and Gynecology, Datta Meghe Institute of Higher Education & Research, Wardha, IND

**Keywords:** poor ovarian reserve, infertility, invitro-fertilization ivf, oocyte quality, platelet-rich plasma/ prp

## Abstract

Platelet-rich plasma (PRP) is a concentrated platelet preparation known for its regenerative properties due to the various growth factors it contains. Its application in the medical field, including dentistry, gynecology, and plastic surgery, has surged. In obstetrics and gynecology, PRP has shown promise in improving low libido, vaginal rejuvenation, ovarian reserve, and endometrial receptivity. This study presents a 29-year-old woman experiencing primary infertility attributed to low levels of anti-Müllerian hormone alongside the presence of asthenozoospermia in her husband’s semen. After failed intrauterine insemination as well as in vitro fertilization (IVF), attempts at laparoscopic PRP treatment were administered before the second IVF cycle to enhance ovarian reserve and quality. The PRP treatment led to an increased follicle count, improved oocyte quality, and a successful pregnancy outcome in the second IVF cycle. PRP treatment promises to be effective in fertility treatments, potentially increasing ovarian reserve, improving oocyte quality, and enhancing successful pregnancy outcomes. This case report highlights its beneficial impact on a couple facing primary infertility, providing hope for patients with similar reproductive challenges.

## Introduction

Anti-Müllerian hormone (AMH) is a glycoprotein produced by Sertoli cells in males and by granulosa cells of the ovary in females. AMH helps in the initial phases of follicular development and prevents premature activation of follicles. It also plays a role in recruiting and selecting follicles that possess the ability to mature during the menstrual cycle [1]. AMH is an important indicator for evaluating ovarian reserve in women. Other indicators for ovarian functions include the patient’s age, antral follicular count, and hormone levels such as follicle-stimulating hormone (FSH), estradiol, and luteinizing hormone (LH). A decrease in oocyte quantity and quality with advancing age is a normal physiologic phenomenon. Sometimes, a decline in ovarian reserve occurs very early in life and can be one of the significant causes of female infertility [2]. Treatment with platelet-rich plasma (PRP) has been shown to be effective in such patients. PRP is concentrated platelets obtained through centrifugation of the patient’s blood. Several growth factors are present in PRP, such as platelet-derived growth factor, vascular endothelial growth factor, insulin-like growth factor, epidermal growth factor, and fibroblast growth factor [3]. These factors stimulate the proliferation of cells, tissue repair, and regeneration; promote angiogenesis; stimulate the migration (chemotaxis) and proliferation (mitogenesis) of different cell types; regulate collagen synthesis; and stimulate immune cells to migrate toward the area of injury.

Nowadays, PRP is commonly employed in the medical field. It is used in orthopedics to treat ligament injuries and osteoarthritis for tissue healing and to reduce pain and inflammation, in dermatology for hair loss treatment and skin treatment, in ophthalmology to promote corneal healing, and in sports medicine to treat injuries [3]. Its use in obstetrics and gynecology has also been explored. PRP has the potential to enhance ovarian reserve and improve oocyte quality in women with low AMH levels [4]. Moreover, PRP is said to help treat female sexual dysfunction. It is believed to enhance sexual response by improving genital blood flow and tissue health. It may help improve vaginal tightness, lubrication, and overall tissue health. PRP has been considered as a potential treatment for stress urinary incontinence and pelvic floor disorders. Studies indicate that PRP enhances endometrial receptivity in women undergoing fertility treatments [5,6]. Due to its multiple functions and applications, PRP has become a valuable therapeutic option for treating infertility. This case report suggests that PRP may have a regenerative effect on ovarian tissue, stimulating cell proliferation and promoting the growth and development of healthy ovarian follicles. These effects could result in increasing the number of viable eggs and improving fertility outcomes in females struggling with diminished ovarian reserve.

## Case presentation

This is a case report of a 29-year-old woman who presented with a complaint of primary infertility seven months ago. Her husband’s age was 31, and they had been married for two years. They had been attempting to conceive for one and a half years. Initially, they sought religious ways to cure infertility, then decided to consult a doctor. There was no significant history or family history. There was no history of previous surgeries. The woman’s age of menarche was 13 years, and she had regular cycles of 29 days with normal flow. The husband, a businessman, had been an active smoker for the past nine years and smoked nine to 10 cigarettes per day.

Investigations

Both underwent a comprehensive investigation. The husband's semen analysis indicated asthenozoospermia, with a sperm count of 20 million/ml and 25% motility. However, his hormonal evaluation, which included tests for testosterone, LH, and FSH, showed no abnormalities. The woman’s evaluation revealed low ovarian reserve, with low AMH levels measuring at 0.2 ng/dl and high FSH levels. The transvaginal ultrasound revealed the presence of three follicles on both the right and left ovaries before the first oocyte retrieval. However, hysterosalpingogram results indicated normal and open fallopian tubes, ruling out tubal factors as a cause of infertility.

Treatment procedure

**Figure 1:**
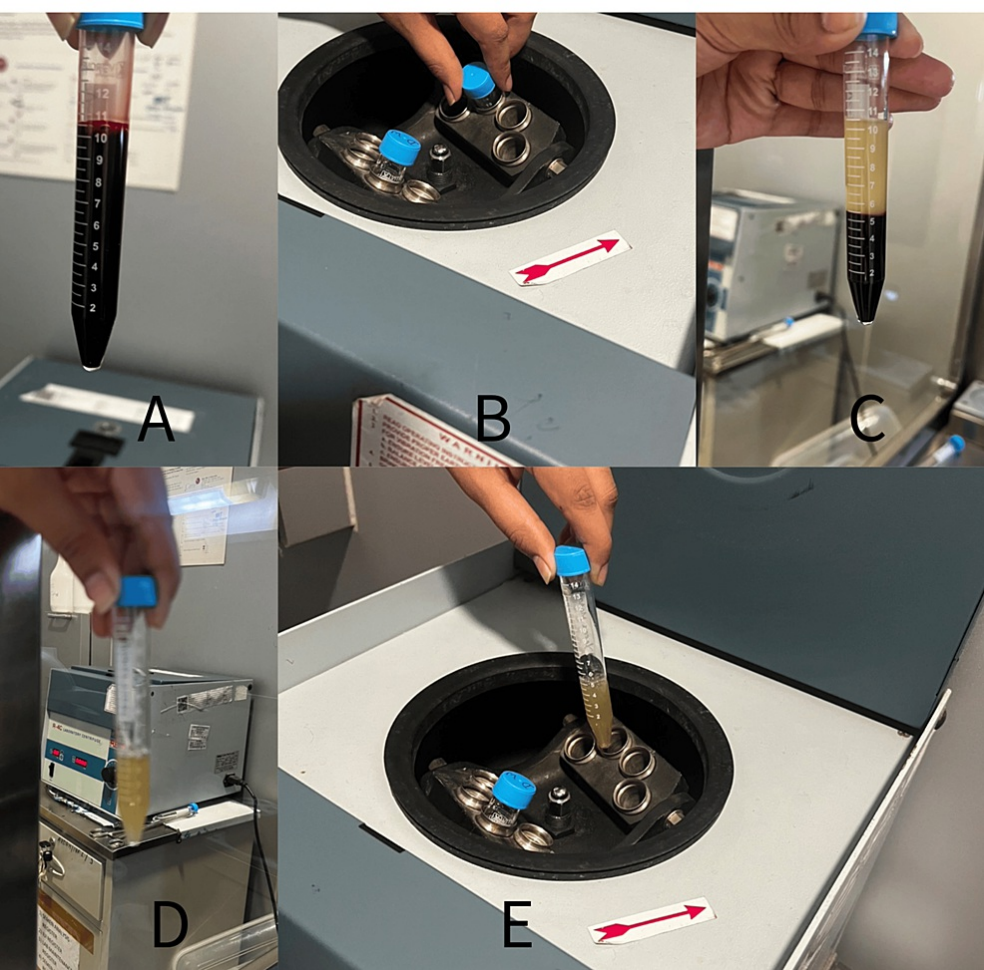
Preparation of PRP. (A) Collection of blood in a sterile tube. (B) Centrifugation of blood at 1,200 rpm for 10 minutes. (C) Separation of blood into three layers. (D) Extraction of the top layer and buffy coat into a separate tube. (E) Further centrifugation at 2,000 rpm for 10 minutes. The original picture was captured by the author of this article. PRP, platelet-rich plasma

Intrauterine insemination (IUI) using the husband’s semen was attempted, but unfortunately, it was unsuccessful. Consequently, the couple opted for in vitro fertilization (IVF) as their next treatment option. After two months, the first cycle of IVF began after the female underwent controlled ovarian stimulation. Antral follicular count was assessed on ultrasound; three follicles were observed in the right ovary, while an additional three were noted in the left ovary. During ovum pick-up, four oocytes were retrieved: one germinal vesicle, two metaphase I (MI), and one MII. The quality of the MII oocyte was not good either. Intracytoplasmic sperm injection was done on the MII oocyte. It got fertilized but degenerated later, so no embryos were formed, and the IVF cycle failed. Two months later, after thorough counseling and consent, the decision was made to proceed with laparoscopic PRP treatment before the second IVF cycle to potentially improve the patient’s ovarian reserve and oocyte quality. PRP was prepared by double centrifugation (Figure 1). To start with, an 11-ml venous blood sample was collected from the woman’s arm in a sterile tube. Then, the blood sample underwent centrifugation at a speed of 1,200 rpm for 10 minutes. As a result, it was separated into three parts: the top had platelets, the middle part had platelets and white blood cells, and the bottom part had red blood cells. After that, the top and buffy coat were carefully separated into a sterile tube. The transferred plasma underwent a second centrifugation for 10 minutes at 2,000 rpm. The top two-thirds of the centrifuged plasma was discarded since it had a lower concentration of platelets. The pellets at the bottom of the tube were mixed and blended with the remaining plasma. At this point, the PRP was considered ready for use. Ultimately, 1.5-2 ml of PRP was instilled laparoscopically in each ovary 10 days after menstruation.

**Figure 2:**
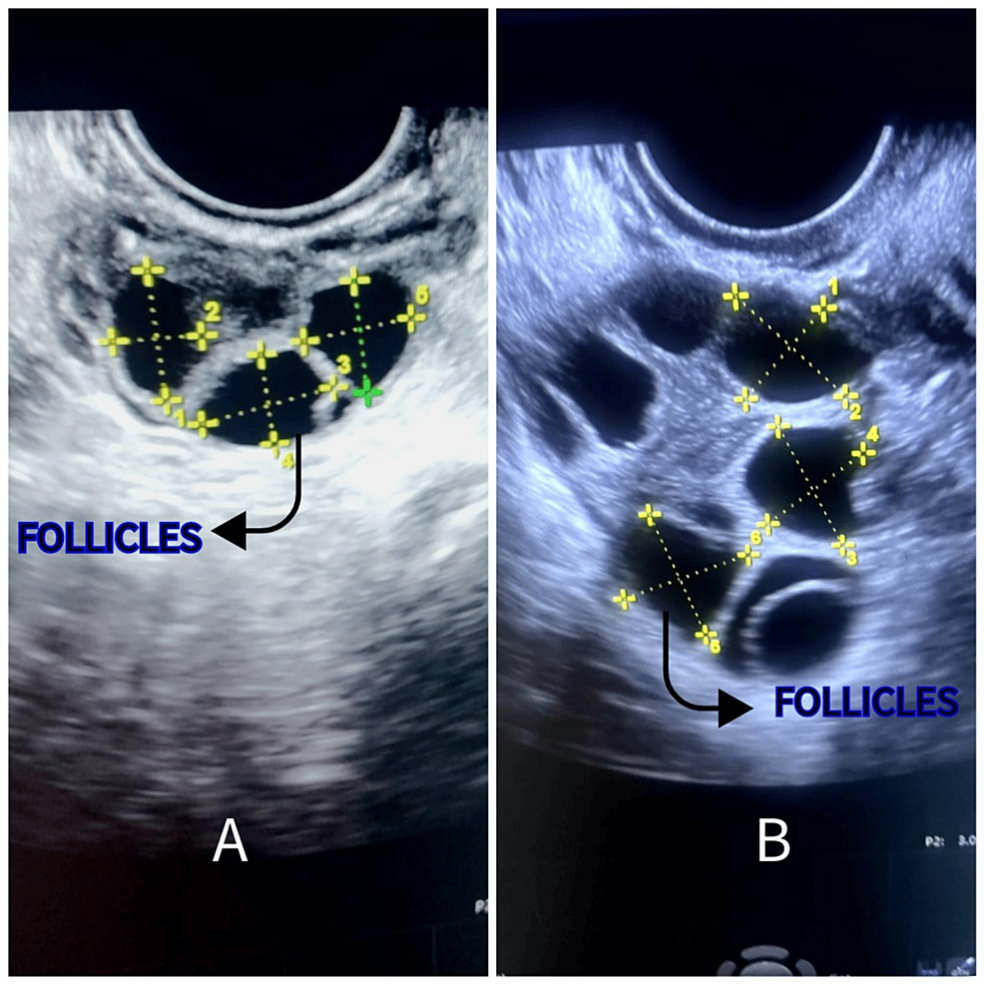
Ultrasonographic image of the right ovary showing follicles. (A) Before PRP therapy. (B) After PRP therapy. The original picture was captured by the author of this article, and informed consent was obtained for publication from the patient. PRP, platelet-rich plasma

**Figure 3:**
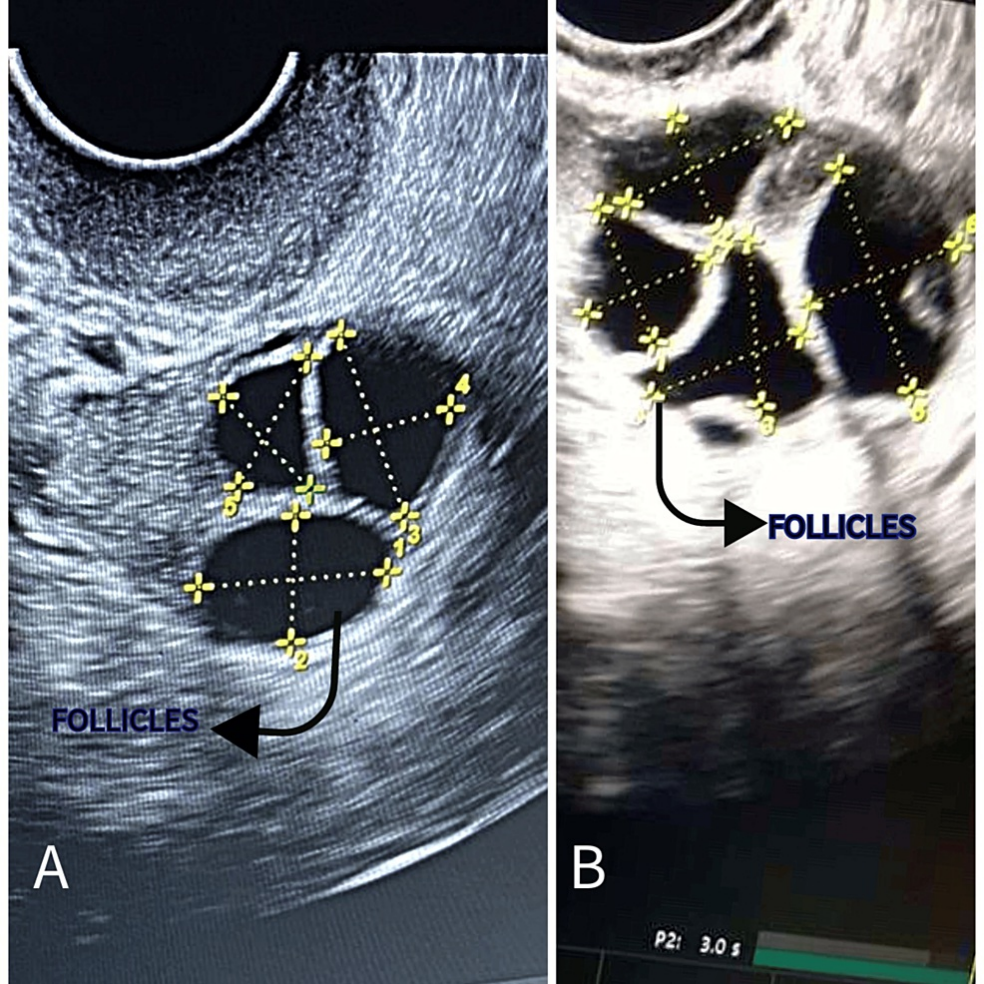
Ultrasonographic image of the left ovary showing follicles. (A) Before PRP therapy. (B) After platelet-rich plasma therapy. The original picture was captured by the author of this article, and informed consent was obtained for publication from the patient. PRP, platelet-rich plasma

In the next menstrual cycle, the female partner received gonadotropin-releasing hormone antagonists. Subsequently, ovarian stimulation was initiated using gonadotropins. The follicular study showed six follicles on the right ovary (Figure 2) and four on the left ovary (Figure 3). On ovum pick-up, eight oocytes were retrieved: two MI and six MII. All six MII were fertilized, and five progressed to the blastocyst stage. Two embryos were transferred (fresh), and three were cryopreserved. After two weeks, the pregnancy test was positive. The couple was overjoyed with the successful outcome of the treatment and expressed their heartfelt gratitude. In this case report, PRP therapy improved ovarian reserve and oocyte quality, resulting in successful implantation and a positive pregnancy outcome.

Follow-up

Following the successful treatment, the woman was advised to undergo regular antenatal check-ups and was prescribed essential prenatal vitamins. Her health and fetal growth were diligently monitored through routine assessments and examinations.

## Discussion

This case report demonstrates that ovarian reserve and oocyte quality were enhanced after PRP treatment. PRP treatment for ovarian reserve is still considered an emerging area of research and is a subject of ongoing investigation. While there have been some studies and case reports suggesting the potential benefits of PRP in improving ovarian reserve and oocyte quality, the overall evidence is limited, and its widespread use is not yet common. A study by Sills and Wood showed increased serum AMH and retrieval of MII after intraovarian PRP treatment [7]. Sfakianoudis et al. reported a remarkable case of a successful pregnancy in a 35-year-old menopausal woman following an intraovarian PRP injection, but she had a miscarriage in the fifth week [8]. Hosseini et al. noticed a notable increase in the formation of early antral follicles when supplementing them with PRP culture for 10 days [9]. This finding led the researchers to conclude that PRP might be a promising and successful method for stimulating follicular development. According to Cakiroglu et al., PRP improves IVF success rates in women with primary ovarian insufficiency (POI) [10]. Aflatoonian et al. investigated the influence of intraovarian PRP injections in females who had POI and poor ovarian response (POR). A total of 47% conceived, and half of those led to live childbirth in women with POR [11]. In an article by Petryk and Petryk, it was found that injecting a concentrated PRP solution into the ovary using ultrasound guidance through the vagina had positive and enduring impacts on reproductive function [12]. In a case report by Hsu et al., the combination of PRP and gonadotropin treatment resulted in a successful pregnancy culminating in a live birth in a 37-year-old woman with POI who previously showed very low responsiveness to external gonadotropin treatment [13]. Similarly, a study by Parvanov et al. demonstrated a notable rise in the count of successfully retrieved MII oocytes as well as the number of blastocysts following PRP treatment. Additionally, they observed enhanced quality of oocytes and embryos in approximately 75% of patients who underwent PRP ovarian treatment [14].

Currently, PRP treatment for ovarian reserve is typically considered experimental, and its application in clinical practice may vary among fertility clinics and healthcare providers. The use of PRP in fertility treatments, including its impact on ovarian reserve, requires further rigorous research and clinical trials to establish its safety, effectiveness, and optimal protocols.

## Conclusions

This case report highlights the role of PRP treatment in ovarian rejuvenation. The successful outcome of this 29-year-old woman’s journey to pregnancy after failed IUI and IVF attempts shows the potential benefits of PRP therapy. It is important to note that individual responses to treatment can vary widely, and this case report is based on a single patient, limiting conclusive interpretation of the findings. By increasing follicle count, improving oocyte quality, and ultimately leading to a successful pregnancy in the second IVF cycle, PRP has demonstrated its potential to enhance fertility treatments. This report offers hope for couples dealing with infertility due to poor ovarian reserve. It still requires further research and clinical applications of PRP in the fields of obstetrics and gynecology. PRP treatment represents an innovative procedure for improving outcomes in fertility treatments.
